# Sleep Health in Human Biology Research

**DOI:** 10.1002/ajhb.70025

**Published:** 2025-03-10

**Authors:** Kristen L. Knutson

**Affiliations:** ^1^ Center for Circadian and Sleep Medicine Northwestern University Feinberg School of Medicine Chicago Illinois USA

## 1 Introduction

One tenet of anthropology is that there are few human universals, and the need for sleep is one of these few human universals. All must sleep, much like we all must eat and breathe, and if we do not sleep, our health is impaired in myriad ways. Despite this universal need, numerous aspects of culture, environment, and biology shape sleep patterns, which result in variations in sleep health among human groups. In my 2012 publication (Knutson 2012), I reviewed the link between inadequate sleep and obesity risk, as well as the associated conditions, diabetes and cardiovascular disease (CVD). To summarize the review, there was both experimental and observational evidence indicating that inadequate sleep, particularly shorter sleep durations, was associated with increased prevalence or incidence of obesity, diabetes, and CVD. In this Commentary, I will revisit the important role human biologists can play in understanding human variation in sleep health, its determinants, and its impact on population/global health.

## 2 Sleep Health and Human Health

**FIGURE 1:**
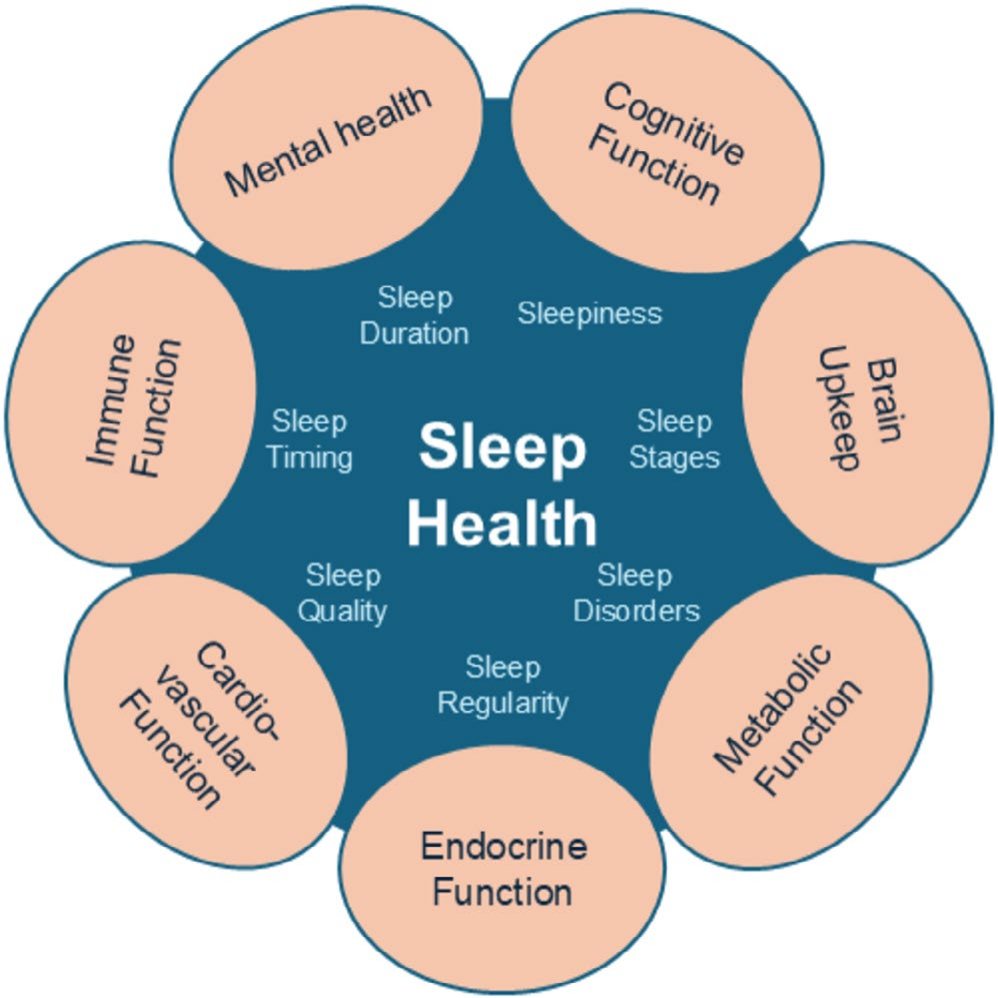
Multidimensional sleep health and associated health domains.

My prior review focused primarily on sleep duration, which has been the most studied characteristic of sleep. However, it is now well recognized that sleep health is a multidimensional phenomenon and that most—if not all—of these dimensions are important for human health (see Figure 1). In addition to the amount of sleep, other dimensions include sleep quality, the timing of sleep (i.e., the clock time), sleep regularity (i.e., sleeping at about the same time from day to day), and daytime sleepiness. The macroarchitecture of sleep, which refers to the different sleep stages, such as rapid eye movement (REM) and non‐REM sleep, as well as the microarchitecture of sleep, which refers to a detailed analysis of the electroencephalogram (EEG), is also an important sleep health characteristic. Finally, the presence or absence of sleep disorders, such as insomnia or sleep apnea, should also be considered.

In its infancy, sleep research focused primarily on brain‐related outcomes, such as memory, cognitive performance or mood disorders. Indeed, it seemed to be assumed that sleep was “by and for the brain” while the rest of the body was ignored. However, the experimental work described in my prior review clearly demonstrated that sleep reaches beyond the brain and impacts our entire physiology. Further, although my prior review focused on cardiometabolic outcomes, substantial research has established a role for sleep in numerous other health domains (see Figure 1), including immune function, which has implications for infectious disease and cancer risk, cognitive function, and brain maintenance, which are linked to Alzheimer's Disease and dementia, and mental health, which can include depression, anxiety or even risk‐taking. Given the broad implications of impaired sleep health for overall human health, sleep should be considered one of the pillars of a healthy lifestyle.

## 3 Human Biology and Sleep Research: An Ideal Combination

Sleep is a biological necessity that is also strongly influenced by behavior, beliefs, and environmental factors. Therefore, sleep patterns will vary among different cultures, regions, and sociodemographic groups. There is substantial opportunity and need for human biologists in the field of sleep research to improve our understanding of both the determinants (both facilitators and barriers) of sleep health and the consequences of poor sleep health for human health and well‐being.

There has been an increase in sleep research among anthropologists and human biologists in recent years. For example, prior to my 2012 review article, between the years of 2002 and 2012, there were 8 articles published in the *American Journal of Human Biology* (*AJHB*) that had “sleep” in the title. Since 2012, there have been 20 articles with sleep in the title in this journal. This pattern mirrors the entire field of sleep research, as interest in sleep has grown across many disciplines and the number of publications from all journals with sleep in the title has grown exponentially (there were > 2 times as many papers published between 2010 and 2024 as there was published between 1900 and 2010). This increase in attention to sleep is exciting and encouraging, particularly in the field of human biology, since this scientific perspective can provide unique insights into sleep from around the world.

Many of these recent articles in *AJHB* also examined the association between sleep and obesity, or associated conditions, and contributed novel findings to this literature. While most research on sleep and obesity has been conducted in high‐income countries, like the U.S., one study examined the association between obesity and both self‐reported sleep duration and sleep quality among older adults from six middle‐income countries: China, Ghana, India, Mexico, Russian Federation, and South Africa (Gildner et al. 2014). They found that shorter sleep was associated with higher body mass indices (BMI) and waist circumference (WC) in both men and women, which was consistent with research in higher‐income countries; however, they also found that higher subjective sleep quality was associated with higher BMI and WC in men from India and China, which is contrary to prior work that found worse sleep quality was associated with higher body size, as described in the prior article (Knutson 2012). The authors attributed their discrepant findings to potential differences in the association between sleep and socioeconomic status (Gildner et al. 2014). Cross‐sectional associations between self‐reported sleep duration and weight status were also examined in over 18,000 adults 65 years and older from a large nationally representative study from China (Zhao et al. 2023), and their results indicated that short sleep (< 6 h/night) was associated with increased prevalence of underweight and long sleep (> 8 h/night) was associated with increased prevalence of obesity in men only. Together, these findings highlight the importance of considering age, gender, and regional differences in the associations between sleep and weight status. These newer papers also included an analysis of nationally representative data from the U.S. (the National Health and Nutrition Examination Survey, or NHANES) that indicated that the association between self‐reported sleep duration and increased mortality risk may be partly mediated by immune factors (Shattuck and Sparks 2022), and these findings further support the interaction between sleep health and immune function, which has implications for a wide variety of diseases and disorders.

There were several recent *AJHB* papers that focused on pediatric populations, which is a vulnerable time for growth and development that can determine trajectories of weight gain. One study was of children aged 6–12 years living in an urban community in Argentina, and they identified parentally reported short sleep (< 8 h/night) as one of the factors significantly associated with overweight/obesity (Orden et al. 2019). A large study of more than 8000 children aged 6–9 years in Portugal collected parental reports of sleep habits and found that boys with inappropriate sleep durations (i.e., < 9 or > 12 h per night) were more likely to be overweight or obese, but no association between sleep duration and prevalence of overweight or obesity was observed in girls (Machado‐Rodrigues et al. 2018), indicating again there may be gender differences in the link between body size and sleep health. A study in Chile examined the associations between parentally reported sleep times and cardiometabolic risk factors in children of either Amerindian Mapuche (*n* = 119) or European ancestry (*n* = 421) (Alvarez et al. 2019). They reported that among the Mapuche children, shorter sleep was associated with higher BMI, larger WC, greater fat mass, and higher systolic blood pressure, while among the European ancestry children, shorter sleep was associated with larger WC, greater muscle mass, and higher systolic blood pressure. Understanding the potential reasons for disparate findings in the two ancestry groups, including sociocultural factors, could provide unique insights into the association between sleep and cardiometabolic health in children, as well as potential mitigating factors.

It is important that we identify barriers and facilitators to good sleep health, and some of these recent papers have contributed to this goal. One paper used data from the U.S. Behavioral Risk Factor Surveillance System (BRFSS) to examine whether insufficient sleep was associated with light pollution, and although effect sizes were small, greater light pollution was indeed associated with insufficient sleep (Patel 2019), which supports light as one potential environmental factor that disturbs sleep. A survey study among college students found that both psychosocial distress and childhood food insecurity were associated with poorer subjective sleep quality (Kopels et al. 2024). The association between food insecurity in childhood and sleep as a young adult has important implications for public policy related to providing access to food among children since such access could have lasting effects on health into young adulthood. A study from Guyana aimed to identify which multilevel factors were associated with adolescent sleep behaviors and reported that living in an urban setting was associated with later bedtimes and older age was associated with both later bedtimes and less sleep, while greater household poverty was actually associated with longer time in bed (Singh and Vitzthum 2019). These findings demonstrated the importance of studying sleep patterns in a wide range of communities since associations observed in wealthy countries or communities are not always generalizable beyond those borders. Finally, a study of military police in Brazil revealed that those officers who underwent elite training obtained less sleep than the non‐elite officers (however both groups averaged less than 7 h/night), and they also found that greater occupational stress was associated with greater daytime sleepiness in the military police officers (Garcia et al. 2024). This study provides novel data for a specific occupation but has broader implications for other occupations where stress levels may be high. Various occupational groups will have unique circumstances that can linger outside of work even into bedtime.

The recent papers from AJHB described above indicate that not all associations observed in the U.S. are generalizable to other regions. For example, children living in poverty in Guyana spend more time in bed, not less, and the men in China and India with better sleep quality have larger BMIs, not smaller. Two studies also reported significant associations between sleep and weight status in males only, which, considering well‐established gender differences in sleep patterns, underscores the need to examine gender roles and practices as important determinants of sleep health as well as potential effect modifiers of the association between sleep health and other health indicators. This work and others have begun to identify possible determinants of sleep health, but much remains to be done.

In addition to the papers discussed above, there has also been novel sleep research conducted outside the industrialized world, providing critical insight into human variation in sleep patterns. One groundbreaking study involved the assessment of sleep via actigraphy in three preindustrial communities: Hadza in northern Tanzania, the Kalahari San in Namibia, and the Tsimane in Bolivia (Yetish et al. 2015). They reported that the range in average sleep duration was more than one hour, i.e., 5.7–7.1 h, and that it varied with season. Further, sleep onset typically occurred approximately 3 h after sunset, while awakening was usually before sunrise. This study provided novel insight into sleep patterns in communities without artificial lights or temperature control. Another study among 120 adult Tsimane forager‐horticulturalists in Bolivia assessed the degree of nightly variation in sleep patterns using actigraphy (Yetish et al. 2018). They found that night‐to‐night variation in sleep duration within individuals averaged 43 min for women and 56 min for men, and variation in sleep onset varied within individuals averaged 39 min for women and 63 min for men. These findings challenge assumptions that “natural” sleep patterns are consistent from night to night. A small study in Madagascar measured sleep using actigraphy in 21 adults and found that sleep duration was shorter, sleep efficiency was lower, and sleep fragmentation was greater on average compared to some samples from industrialized countries, which indicates worse sleep health in this community (Samson et al. 2017). Finally, an examination of sleep among different primate species revealed that humans have shorter sleep durations than predicted for our body mass but more REM sleep (Nunn and Samson 2018), which suggests that REM sleep in particular may have played an important role in human evolution and brain development. A study in Mozambique compared sleep measured via actigraphy among residents living in one urban (Milange) and one rural (Tengua) town (Beale et al. 2017). Average sleep duration did not differ between towns, but sleep quality was poorer among residents in the rural town, while bedtimes were approximately 1 h later on average for those in the more urbanized town. A similar study in the Amazon region of Brazil compared actigraphically measured sleep among 22 residents in a rural area and 20 residents in a town (Martins et al. 2020). They also found that residents of the town had later bedtimes, but they also had shorter average sleep duration than those living in the rural area. Altogether, this research has filled gaps in our understanding of variations in sleep patterns in different societies and in relation to urbanization.

### 3.1 Possible Determinants of Sleep Health

We have discussed above several possible determinants of sleep health, including age, gender, and environmental factors such as light; however, there are likely many more. Many of these potential determinants of sleep health will vary among communities and therefore are of particular interest to the field of human biology. Determinants of sleep health could be cultural, social, environmental, or biological, and a few examples of each are described below. These examples are not an exhaustive list, nor are they independent of each other (there could be interactions among them).

Cultural determinants of sleep health include cultural beliefs about sleep, including where, why, when, and with whom one sleeps. Worthman and Melby first described the ecology of sleep in 2002 as an important gap in anthropological research (Worthman and Melby 2002). This ecology includes all the conditions under which people sleep and is an important factor underlying human variation in sleep patterns. The sleep environment includes where one sleeps (e.g., on a bed, on a mat, in a hut or walled structure), what materials are used (e.g., pillows, blankets), and any other devices present in the sleeping area (televisions, fans, etc.). In addition, the value of sleep will inform how much effort is put into supporting good sleep health; if sleep is not considered important for health and well‐being, little to no effort will be expended to ensure good sleep. Further, expectations about sleep have the potential to impact sleep. For example, if one believes that falling asleep and staying sound asleep for 8 h continuously is what defines healthy sleep (it does not), then this person may become anxious when they wake in the night, which will make it more difficult to fall back to sleep. Other sleep practices, such as napping, or the individuals with whom you sleep (spouse, child) can also influence sleep health. Despite this lengthy list of possible cultural determinants of sleep health that can shape sleep patterns, only a few studies have been conducted to understand beliefs and practices pertaining to sleep. Yetish and McGregor (Yetish and McGregor 2019) reviewed sleep in hunter‐gatherer societies, including descriptions of factors that influence sleep, including the sleep environment, such as sleeping surfaces and dwellings, nighttime food acquisition, rituals, or environmental factors (temperature, moonlight).

There are additional social and behavioral factors that impact sleep health, including one's gender identity and associated roles and expectations. Greater exposure to psychosocial stressors can impair sleep health (Akerstedt et al. 2007). As mentioned above, one's occupation can influence sleep, particularly working night shifts or having a physically demanding job. Health behaviors such as smoking, alcohol use, diet, and physical activity can all affect sleep. For example, both smoking and alcohol use are associated with worse sleep health (Hussain et al. 2022), while exercise appears beneficial to sleep (Yang et al. 2012). Finally, there are several factors that are beyond the individual level that influence sleep health, including interpersonal interactions, such as experiences of discrimination or social support, as well as institutional‐level factors, such as healthcare systems, political systems, or religious institutions (Jackson et al. 2020). Some of these factors could facilitate obtaining good sleep while others will be barriers to good sleep.

Several environmental factors influence sleep and clearly vary by region of the globe. Environmental conditions can even vary within a few miles in a single city, particularly with respect to the built environment. Also, as climate change continues to reshape everyone's environment, and not for the better, extreme environmental conditions such as excessive heat or storms will impair sleep health (Gaston et al. 2023). Another important environmental factor for sleep health is access to and use of artificial light (Mead et al. 2023). Light is the strongest synchronizer of our internal biological clock (Dibner et al. 2010), and exposure to light during the biological night can disrupt this clock and associated rhythms, such as sleep regulation. Exposure to light at night is only partially under one's control, such as the use of light in one's home, however, bright streetlights in certain urban areas could increase exposure to light at night and impair sleep, particularly in under‐resourced communities. Similarly, the temperature and humidity in one's sleeping area are not always under one's control but can influence sleep. Temperatures that are too hot or too cold will make sleep more difficult (Baniassadi et al. 2023), as will high humidity, but not every home has access to air conditioning, dehumidifiers, or even adequate heat. Noise also disrupts sleep (Hume et al. 2012; Kageyama et al. 2016) and there are several potential sources of noise, some from within the home (leaving the TV on, noisy household members) and outside the home (e.g., traffic, animals, sirens, neighbors). Air quality could also impair sleep (Basner et al. 2023), particularly in those who are more vulnerable, such as those with asthma. Finally, there are other neighborhood characteristics that may be important, such as building quality, proximity to transit areas, and green space. All of these environmental characteristics warrant further research.

Finally, there are biological factors that impact sleep, including age and sex. Sleep is also known to vary across the menstrual cycle, during pregnancy, and during the menopausal transition (Izci‐Balserak et al. 2018; Jones et al. 2018). Specifically, sleep quality is often worse during the premenstrual phase and during menstruation, often due to menstrual symptoms such as cramps (Alzueta and Baker 2023). During late pregnancy, sleep continuity is often disrupted, and sleep‐disordered breathing is more common, and these sleep disruptions are likely due to frequent urination, physical discomfort, leg cramps, and restricted space for the lungs (Izci‐Balserak et al. 2018). Finally, subjective sleep quality declines during the menopausal transition, often due to associated symptoms like feeling too hot (Jones et al. 2018). Age is also often associated with a decline in sleep quality, and this association seems to vary between men and women, where older men have worse sleep quality than older women (Taporoski et al. 2024). Other biological factors include several hormones that affect sleep. In particular, cortisol and adrenergic hormones can impair sleep (Born et al. 1989) while melatonin helps facilitate sleep (Ferracioli‐Oda et al. 2013). Acute and chronic illness, as well as some associated medications, could also impact sleep. Indeed, there is a bidirectional relationship between infection, the immune system, and sleep whereby impaired sleep can impair immune function, but infection and the immune response can alter sleep (Feuth 2024). Chronic illness is also often associated with poorer sleep quality due to pain, discomfort, or other symptoms. Finally, some genes are linked to sleep (Lane et al. 2023), and these could provide insight into mechanisms and potential targets for therapy when warranted.

Overall, there are several possible barriers to good sleep health, such as poor environmental or social conditions, and these have important implications for understanding sleep health disparities. A workshop sponsored by the National Institutes of Health summarized the “causes and consequences of sleep health disparities” (Jackson et al. 2020). Fewer facilitators for good sleep health have been identified (other than eliminating the barriers), but they likely exist, including social support, valuing sleep, and realistic expectations about sleep. If we want to develop methods to improve sleep health, identifying these facilitators and an important step. However, we must be aware of the barriers and consider the reality of competing priorities.

### 3.2 Competing Priorities

In evolutionary medicine, scholars have discussed trade‐offs in the allocation of resources to reproduction, growth, or maintenance (Stearns 2005); however, individuals also make trade‐offs with respect to time. There are only ~24 h in a day, yet there are many activities that need to be accomplished each day. Individuals must prioritize these activities, and many of these activities will replace sleep. Competing priorities could include obtaining resources for oneself and dependents, which would include employment in some societies, child or elder care, or household activities as possible examples. Because humans have the ability to ignore the drive for sleep (up to a point), sleep can be replaced by these competing priorities. However, how, why, and when these trade‐offs occur between sleep and other activities have not been thoroughly examined, and they may provide meaningful insight into barriers and facilitators to good sleep health.

## 4 Future Directions

Understanding the cultural milieu in which sleep is experienced and managed is critical to developing tailored interventions to improve sleep health; what works in one place will not necessarily work in another. Therefore, we need to fill these research gaps on the cultural beliefs and practices related to sleep and how they are associated with sleep health in various communities. The perspective of the community members themselves is also an important but missing piece, and one human biologists and anthropologists are well poised to obtain.

Further, engaging the community members in the development of these interventions will further ensure their success. Community‐based participatory research is an established approach to successful research employed by human biologists and anthropologists for decades. Therefore, a human biology approach is of great value to understand the link between sleep health and overall health as well as the development of methods to improve sleep.

Finally, given the link between sleep health and other health domains, sleep health may play an important role in health disparities among socioeconomic, racial, or ethnic groups. Many of the determinants of sleep health described above are particularly salient when considering health equity. Future work needs to continue to elucidate the role of sleep in health disparities.

## 5 Conclusions

Sleep health is associated with many other health outcomes, including cardiometabolic health, immune function, cognitive function, and mental health. The list of individual, interpersonal, and institutional factors that can shape sleep health is extensive. Research on sleep and health has been increasing, including in human biology, but many questions remain. Since sleep is a biological phenomenon heavily influenced by behavior and environment, promoting sleep health will be a complex process, and human biologists can provide a critical perspective to answer these questions. Over the next 50 years of the Human Biology Association, I expect that sleep will become an even more common component of human biology research, particularly since all humans sleep and sleep is an important pillar of human health.

## Data Availability

Data sharing is not applicable to this article as no new data were created or analyzed in this study.
